# Reconstructing Nonparametric Productivity Networks

**DOI:** 10.3390/e22121401

**Published:** 2020-12-11

**Authors:** Moriah B. Bostian, Cinzia Daraio, Rolf Färe, Shawna Grosskopf, Maria Grazia Izzo, Luca Leuzzi, Giancarlo Ruocco, William L. Weber

**Affiliations:** 1 Department of Economics, Lewis and Clark College, Portland, OR 97219, USA; mbbostian@lclark.edu; 2 Department of Computer, Control and Management Engineering A. Ruberti (DIAG), Sapienza University of Rome, 00185 Rome, Italy; mariagraziaizzo@gmail.com; 3 Department of Applied Economics, Oregon State University, Corvallis, OR 97331, USA; rolf.fare@oregonstate.edu; 4 Department of Economics, Oregon State University, Corvallis, OR 97331, USA; shawna.grosskopf@oregonstate.edu; 5 Center for Life Nano Science, Fondazione Istituto Italiano di Tecnologia (IIT), 16163 Rome, Italy; Giancarlo.Ruocco@roma1.infn.it; 6 Soft and Living Matter Lab, Institute of Nanotechnology, 00161 Rome, Italy; luca.leuzzi@roma1.infn.it; 7 Department of Physics, Sapienza University of Rome, 00185 Rome, Italy; 8 Department of Accounting, Economics and Finance, Southeast Missouri State University, Cape Girardeau, MO 63701, USA; wlweber@semo.edu

**Keywords:** networks, data envelopment analysis, entropy, generalized multicomponent Ising model, Georgesçu-Roegen flows and funds model, Bayesian statistics, knowledge production

## Abstract

Network models provide a general representation of inter-connected system dynamics. This ability to connect systems has led to a proliferation of network models for economic productivity analysis, primarily estimated non-parametrically using Data Envelopment Analysis (DEA). While network DEA models can be used to measure system performance, they lack a statistical framework for inference, due in part to the complex structure of network processes. We fill this gap by developing a general framework to infer the network structure in a Bayesian sense, in order to better understand the underlying relationships driving system performance. Our approach draws on recent advances in information science, machine learning and statistical inference from the physics of complex systems to estimate unobserved network linkages. To illustrate, we apply our framework to analyze the production of knowledge, via own and cross-disciplinary research, for a world-country panel of bibliometric data. We find significant interactions between related disciplinary research output, both in terms of quantity and quality. In the context of research productivity, our results on cross-disciplinary linkages could be used to better target research funding across disciplines and institutions. More generally, our framework for inferring the underlying network production technology could be applied to both public and private settings which entail spillovers, including intra- and inter-firm managerial decisions and public agency coordination. This framework also provides a systematic approach to model selection when the underlying network structure is unknown.

## 1. Introduction

Economic production often results from complex systems of inter-connected production processes, forming a unified network production technology. Data Envelopment Analysis (DEA) methods have long been used to estimate production technologies and measure relative performance. Network DEA (NDEA) models provide a generalization to assess the performance of complex systems, in which separate production subtechnologies are linked. Network linkages include intermediate output/input relationships [1,2,3,4], as well as dynamic connections across time [5,6] and location [7,8,9], and two-way feedback effects between subtechnologies [10]. Chen et al. [11] consider potential pitfalls related to subtechnology efficiency, frontier projection, and the treatment of intermediate quantities when using envelopment methods. Chen et al. [12] and Cook et al. [13] review the literature for two-stage network models. Kao [14] provides a more recent, comprehensive review of the NDEA literature. Several handbooks [13,15] cover topics related to practical implementation of NDEA.

While NDEA models can accommodate a large number of sub-activities and interactions, the curse of dimensionality requires that the number of sub-activities/interactions be relatively small compared to the number of observations for NDEA to discriminate differences in performance. This poses a tradeoff between better structurally representing system dynamics and better assessment of relative performance. To avoid the curse of dimensionality, we estimate a relatively parsimonious NDEA model and then use the resulting performance estimates as inputs into an entropy-based statistical model that allows recovery of a wider set of network interactions.

Uncertainty surrounding model selection poses another common limitation for NDEA. Kao [16] argues that it is not possible to *a priori* choose which network model to apply in a given empirical context. Most existing studies analyze productivity networks in a descriptive way, without considering them in a statistical framework. As a result, the network structure is generally assumed and not estimated. Trinh and Zelenyuk [17] offer one exception, proposing a bootstrap-based comparison between average DEA-NDEA efficiency scores and their distributions, but without questioning the network structure, i.e., assuming the NDEA structure.

We propose a statistical-based approach to reconstruct (infer) the network’s structure, for nonparametrically-estimated productivity frontier models. We develop a Bayesian framework that relies on recent Pseudo-Likelihood techniques introduced in the physics of complex systems [18,19,20,21] for estimation. Our approach can be considered semi-parametric because it bases inference of the network structure on a parametric Bayesian approach generalized multicomponent spin model) to make inference for nonparametric NDEA productivity networks.

Up to now, statistical inference for NDEA models has been constrained by the lack of standard inferential tools needed to evaluate complex systems. We provide a reasonable and robust inferential approach that allows us to reconstruct productivity networks empirically, starting from the observed NDEA data. The expected new insight gained by applying our methodology is then to infer the productivity network structure from the data instead of assuming it *a priori*. Although our inferential approach is parametric and relies on various parametric assumptions and Bayesian statistics, its foundation is laid by Georgesçu-Roegen [22] in which the economic process follows the second law of thermodynamics—the entropy law.

Our work also closely relates to recent developments in the econometrics of information [23,24,25], statistical inference, and machine learning [26,27]. By including work from information science and machine learning methods borrowed from the physics of complex systems, we fill an existing gap in the NDEA literature related to the choice of the network structure, described as an open issue in recent books on the subject [15,16]. Ours is also a timely contribution, as the diffusion of computational power of computers permits the implementation of new inferential tools based on machine learning techniques.

Our empirical illustration examines cross-country and cross-discipline knowledge production spanning 16 STEM (Science, Technology, Engineering, Math) fields and 17 years. We estimate research linkages across individual fields, both within and across countries over time, to assess research performance in both quantitative and qualitative terms. Our key insight from the application is that while estimated efficiency measures for research output exhibit generally low correlations across disciplines, we find that many of these disciplines exhibit relatively high interdependencies. Simple correlation measures fail to capture underlying structural relationships connecting research disciplines.

Our framework for inference extends previous applications that model knowledge networks through NDEA (see [6,28,29,30,31]). Daraio [32] shows that the complexity of research productivity and the expansion of networks in economics provides impetus for the search for new and more general models of the production process. Our application extends Daraio et al. [33] to estimate scientific knowledge productivity, offering a more general network model that accounts for the complexity of research production.

The paper unfolds as follows. In the next section, we illustrate the main features of the economic model. Section 3 presents the axioms of the underlying DEA models and their connection to those of the general NDEA model. Included is a schematic of the general types of structures of NDEA models. This section also introduces the Georgesçu-Roegen [22] flow and funds model (GRFF) and its connection with our NDEA model. Section 4 illustrates the connection between the statistical approach proposed and the GRFF model. Section 5 introduces the knowledge production network that we estimate, including a schematic of the possible cross-disciplinary links that our statistical second stage estimates can reveal. Next is a description and summary statistics of the data, followed by the outlines of the alternative parsimonious NDEA models we estimate in the first stage. A formal statement of the NDEA problem objective and constraints follows. Descriptive analyses of the first stage productivity models are included, followed by the main results of the application of the second stage to our knowledge production. The final section provides a discussion of our approach and results. We include two technical appendices: Appendix A contains an introduction to the Ising spin glass model while Appendix B provides additional technical details on the Pseudo-likelihood approach. We also include a more detailed summary of our data in Appendix C.

## 2. The Economic Model

The axiomatic production theory behind this paper and described in Section 3, is found in [1,34] and [35]. Färe and Grosskopf [3] introduce the concept of NDEA and extend the axioms to a network setting. Section 3.2 highlights the correspondence of the axiomatics of NDEA with the representation of the production process with flows and funds a la Georgesçu-Roegen. This correspondence yields a new, more general framework for modeling production processes by integrating the production process, information theoretic approaches to econometrics, machine learning and statistical inference from the physics of complex systems.

As described by Prieto and Zofio [4], NDEA within an input-output model (for an introduction and a deep overview see [36]) allows us to gauge potential productivity gains by comparing technologies corresponding to different “economies”. Such models represent a network where different sectoral nodes use primary inputs (endowments) to produce intermediate input and outputs (according to sectoral technologies). In graph theory terms, in an input-output model, each sector (industry) is represented by a node and each flow of intermediate inputs and outputs is represented by a link. Hence, it is possible to optimize primary input allocation, intermediate production and final production using NDEA. This framework allows us to model the different sub-technologies corresponding to alternative production processes, to assess efficient resource allocation among them, and determine potential output gains that could be realized by reducing inefficiencies. In this setting, we use statistical inference to estimate the chains/path connections within and between nodes in order to reveal the underlying structure of the input-output system (see [36], p. 675).

## 3. Axiomatics of DEA Network Models

### 3.1. DEA

#### 3.1.1. Basic Axioms

NDEA is widely used in economics and operations research to assess efficiency and productivity in complex technologies or systems. As of 23 November 2020 Google Scholar identifies 173,000 articles identified under ‘Network DEA’ between 1996 and 2020. It is an extension of Data Envelopment Analysis (DEA) which was introduced by Charnes, Cooper and Rhodes [37]. As its name suggests, DEA envelops input-output data to identify the best practice ‘frontier of technology’ in the sample data. Individual data points are compared to that best practice frontier to determine relative performance.

A well-known issue associated with DEA is the curse of dimensionality: adding more inputs and outputs to the model requires more data to discriminate performance. DEA as an estimator has a slow rate of convergence, making statistical inference difficult. This difficulty is inherited by and compounded in NDEA. NDEA has the additional issue of inference concerning the structure of the more complicated model structure, which connects multiple subtechnologies. This can be structured in many ways, i.e., there are many potential models. This motivates our research question: is there a statistical way to infer the structure of the network model? And can we use that as guidance in model selection when we are forced to specify fairly parsimonious network models due to the lack of data and curse of dimensionality. Our contribution is the derivation and application of just such a statistical approach to structure inference and model selection for NDEA.

NDEA models are a generalization of the basic DEA or activity analysis models of technology and efficiency; they are often referred to as looking inside the black box technology assumed in more reduced-form DEA efficiency models. NDEA can be used to model production processes when the choice of inputs/outputs in one period affects what can be produced in subsequent periods. In addition, NDEA can be used to model production processes where intermediate products are produced in one stage of production and are then used to produce final outputs in another stage. They are also useful when production entails spillovers that can enhance (as in our knowledge production application) or detract from production by other producers/DMUs.

Following [1], we show that the axiomatic underpinnings are similar to those of the standard DEA-estimated technology. Notationally, for inputs, $x\in {ℜ}_{+}^{G}$, and outputs, $y\in {ℜ}_{+}^{M}$, we define the graph of technology, or production set, which relates inputs to outputs:

GR={(x,y):xcanproducey}.

Depending on the problem at hand, we can model the technology equivalently in terms of the input set:

L(y)={x:(x,y)∈GR}

or the output set,

P(x)={y:(x,y)∈GR}.

For estimation, activity analysis (DEA) models generally employ a set of linear constraints on the inputs and outputs to construct the so-called piecewise linear frontier of the technology set, whether GR,L(y) or P(x), in accordance with the basic axioms of production theory (listed below and following [35]).

Following the terminology coined by Charnes, Cooper and Rhodes [37] let there be *D* activities or Decision Making Units (DMUs) i.e., γ=1,…,D. Each activity (DMU) has an associated input–output vector $\left(x^{\gamma },y^{\gamma }\right)=\left(x_{\gamma 1},\ldots ,x_{\gamma G},y_{\gamma 1},\ldots ,y_{\gamma M}\right)$. Kemeny, Morgenstern and Thompson [38] relaxed the von Neumann [39] axioms that all inputs/outputs for each DMU be strictly positive and proposed the following non-negativity conditions on the input and output data, where the data are sometimes referred to as ‘coefficients’. These conditions essentially require that the data matrix be of full rank. These include:

- $\sum_{m=1}^{M}y_{\gamma m}>0,\;\;\gamma =1,\ldots ,D,$ each DMU produces some output;
- $\sum_{\gamma =1}^{D}y_{\gamma m}>0,\;\;m=1,\ldots ,M,$ each output is produced by some DMU;
- $\sum_{\gamma =1}^{D}x_{\gamma g}>0,\;\;g=1,\ldots ,G$ each input is used by some DMU;
- $\sum_{g=1}^{G}x_{\gamma g}>0,\;\;\gamma =1,\ldots ,D$ each DMU uses some input.

If these assumptions are satisfied, then following [35], Färe and Grosskopf [1] show that the basic activity analysis (DEA) technology, here specified as an output set:

(1) $$\begin{array}{cc} P(x)=\{y: & y_{m}\;≦\;\sum_{\gamma =1}^{D}\lambda_{\gamma }y_{\gamma m},\;\;m=1,\ldots ,M,\\ & \sum_{\gamma =1}^{D}\lambda_{\gamma }x_{\gamma g}\;≦\;x_{g},\;\;g=1,\ldots ,G,\\ & \lambda_{\gamma }\;≧\;0,\;\;\gamma =1,\ldots ,D\} \end{array}$$

satisfies the axiom set below, which provides a minimal set consistent with neoclassical production theory. We note that the λ variables are so-called intensity variables which serve to ‘construct’ the piecewise linear frontier of the technology/output set. A typical DEA application using the constraints in P(x) above (1), seeks to maximize outputs for each DMU, subject to the input and output constraints based on the entire data set. This yields an efficiency score for each DMU, where a value of unity signals best practice performance.

The basic production axioms include:

A.1 $0\in P\left(x\right),\;\;\forall x\in {ℜ}_{+}^{G},\;\;y\notin P\left(0\right),\;\;y\geq 0$. (inactivity is feasible);
A.2 x∈L(y),λ≧1⇒λx∈L(y) (weak disposability of input);
A.2S $x\;≧\;x^{o}\in L\left(y\right)\;\;\Rightarrow x\in L\left(y\right)$ (strong disposability of input);
A.3 y∈P(x),0≦θ≦1⇒θy∈P(x);
A.3S $y\;≦\;y^{o}\in P\left(x\right)\Rightarrow y\in P\left(x\right)$ (strong disposability of output);
A.4 $\forall x\in {ℜ}_{+}^{G},\;\;P\left(x\right)$ is bounded;
A.5 The graph is a closed set.

These are minimal axioms consistent with neoclassical production theory. A.1 allows for inactivity, A.2–A3S describe feasible constraints on inputs and outputs, imposed through the respective inequalities imposed in the input and output constraints in P(x). A.4 requires that DMUs cannot produce unlimited output with given inputs, and A.5 requires that the graph technology set contain its boundary, which then serves to identify best practice.

In addition, it is often convenient to assume convexity of the input and output sets. The general result is that if each subtechnology in the network satisfies the Kemeny et al. conditions [38], then the network satisfies the axioms above. Similarly, if each subtechnology exhibits constant returns to scale, then the network also exhibits constant returns. We note that this holds for directed networks.

#### 3.1.2. What Makes a Network?

**Figure 1:**
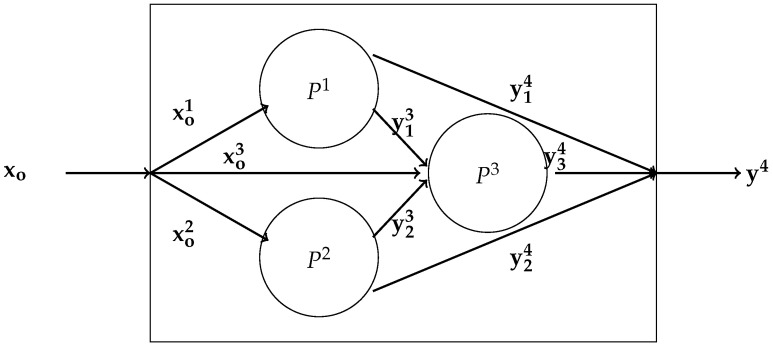
Three node network with input source and output sink.

We introduce the general structure of the network model with a figure first introduced by [40], which illustrates several types of networks. See also [41]. The box in the figure represents the basic DEA models with exogenous inputs $x_{o}$ entering the ‘black box’ producing outputs $y^{4}$ exiting the black box technology. Ignoring the interior of the box would be consistent with the DEA technology described by the linear constraints in (1). The network model allows specification of multiple processes or subtechnologies. Here we assume that there are three sub-technologies

$$P^{1},P^{2},P^{3}$$

organized as in Figure 1, where outputs from $P^{1}$ and $P^{2}$ enter $P^{3}$ as inputs.

We extend this model to include a source, ‘o’, which distributes inputs to the network and a sink, ‘4’, which collects the network outputs. The notation identifies the source of the variable with a subscript and the destination with a superscript. So $x_{o}^{i},i=1,2,3$ means that input $x_{o}$ is distributed to the three subtechnologies, and we have

$$x_{o}\;≧\;x_{o}^{1}+x_{0}^{2}+x_{o}^{3}.$$

Similar notation is used for outputs, so $y_{2}^{3}$ means that outputs from $P^{2}$ are inputs into $P^{3}$. The final output is

$$y=y_{1}^{4}+y_{2}^{4}+y_{3}^{4},$$

with the appropriate choice of dimension of the output vectors. This schematic includes the possibility of parallel subtechnologies or processes such as $P^{1}$ and $P^{2}$, as well as sequential sub-technologies which could be linked through time providing a basis for a dynamic network or supply chain, echoing the earlier work of Georgesçu-Roegen.

A formal mathematical statement of the network problem we solve in our illustration is deferred to Section 5.

### 3.2. Connection with Georgesçu-Roegen’s Flows and Funds Model

The network model analyses the joint actions of different activities within a process. Our theoretical framework allows us to analyze and represent production processes much such as the Georgesçu-Roegen Flows and Funds model (hereafter GRFF model) as subtechnologies which are connected to form the broader network via a maximum entropy condition. In this section, we show how the GRFF Model bridges the axiomatic of NDEA and estimation techniques based on complex systems.

**Figure 2:**
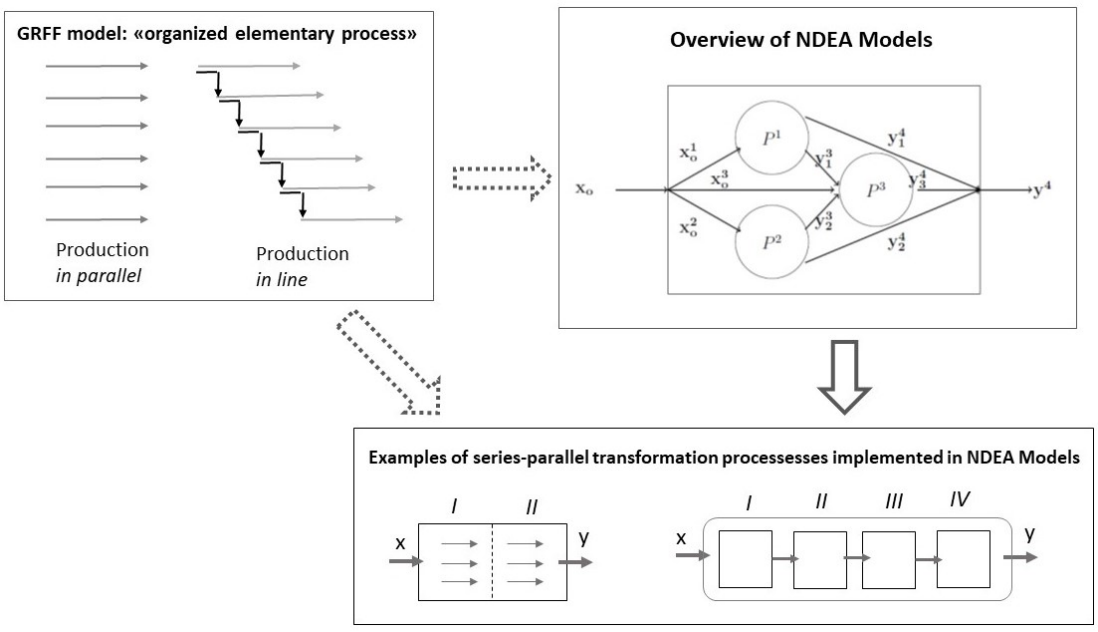
Connections among GRFF Model, our Axiomatics of NDEA and examples of NDEA models.

NDEA models use the structure of networks to model production processes. Georgesçu-Roegen in the 1970s proposed a production model based on “organized elementary process” which can be in line or in parallel. We observe here that this production element is implicitly used in NDEA models. The “organized elementary process” of the GRFF model is the main ingredient or kernel of the axiomatics of NDEA introduced in Section 3 and of the transformation processes modelled in the NDEA literature. We think we are the first to point out the correspondence illustrated in Figure 2.

Figure 2 contains three panels. The North-West panel shows the elementary unit of the Georgescu-Roegen production model, the so-called “organized elementary process” which can be of parallel or in-line production. The North-East panel illustrates the Network DEA models that we presented in the previous section, characterized by both parallel ($P^{1}$ and $P^{2}$) and in-line production processes (such as $P^{1}$ and $P^{3}$ or $P^{2}$ and $P^{3}$). The South panel shows two examples of processes modeled in NDEA models. The model on the left illustrates a two-stage production process in parallel while the model on the right shows a four-stage production process in line. As mentioned above, GRFF’s “organized elementary process” is implied in the North-East and South panels and for this reason, in Figure 2, we reported in dashed form the arrows from the North-West panel towards the other two panels. In this way, we highlight how GRFF’s “organized elementary process” is implicitly contained in the other two panels.

The schematic representation of NDEA and the possibility of including both parallel and sequential sub-technologies can be linked then to the GRFF model. Generally (see e.g., [42]), the model takes into account the actual characteristics of production elements and processes, such as, indivisibility, complementarity, tacitness and heterogeneity of productive knowledge.

In the GRFF model, a flow is an input or an output that enters or exits from a process (for example, energy, water, software, loom, computer, etc.). A fund provides its services to several processes that occur over time (for example, worker, software, land, loom, computer, etc.). A distinction is made between the agents of production processes and the services that they provide. Activities consist of different operations which require the performance of one or more elementary tasks. An elementary task is an operation which, by definition, is not further divisible (for instance, loading or unloading an intermediate product or cutting a piece of fabric). The GRFF model can be implemented both at the microeconomic level, considering individual case studies, and at the macroeconomic level, analysing a set of production units in different sectors of activity.

The GRFF model allows the analytical representation of the organization of production processes including the organization and time dimension of production processes. The formulation of the network production technology presented in Section 3 is an implementation of the GRFF model. The GRFF model may also be connected to the neo-Schumpeterian interpretative framework of production of new processes by means of creation and diffusion of knowledge [43], in which there is an interplay between capabilities, transactions and scale and scope to explain the boundary and the competitiveness of the analyzed units [44].

## 4. The Statistical Model

### 4.1. Maximum Entropy and Georgesçu-Roegen

The principle of maximum entropy serves as the foundation of the theory of inference [45], providing the statistical mechanics to reconstruct probabilistic information from incomplete data. Physical systems evolve spontaneously and possess stability characteristics at equilibrium, which is characterized by the value of maximum entropy. The key to the application of the principle (see [45]) is associating to a probability density function (pdf) an entropy function that measures the dispersion or uncertainty with which the occurrence of possible events are expected. This allows us to introduce constraints, based on our knowledge of the system, that can be treated with the formalism of Lagrange multipliers (see Section 4.2 for more details).

Generally, entropy may be interpreted as: (i) a measure of disorder in a system, (ii) a measure of our ignorance of a system, and (iii) an indicator of the irreversible changes in a system [46].

The Austrian school posits the economy as a complex system that is the outcome of uncoordinated individual behavior [47,48]. In such systems, equilibria do not always refer to a “stationary state” but instead are related to the concept of attractors. An attractor is a deterministic sequence of states which are cyclically visited by the system. As such, it becomes impossible to fully understand macro processes by examining individual behavior. Although representatives of the Austrian School had a skeptical regard to the use of mathematical tools in economics [49], their ideas can be expressed through the lense of statistical thermodynamics or the theory of information (see also [50]).

In chapter VI of his 1971 book [22], Georgesçu-Roegen describes the introduction of statistical mechanics and highlights the connection of economic processes with the second law of thermodynamics, i.e., the entropy law.

We draw the connections here between statistics, economic productivity, and physics of complex systems. As is well known, the correlation between two variables can be influenced by other confounding factors, and does not imply a direct causal effect of one variable on another. On the other hand, interactions or interdependencies refer to strict relationships between variables, allowing us to describe the impact of the variation of one variable on another. Economic productivity models can be used to analyze these interdependencies in a production process, as well as the interconnections between different economic sectors. Physics investigates the interactions between particles in order to analyze direct and reciprocal effects.

We finally highlight the fact that in information theory the maximum entropy problem can be reformulated as a minimum cost of coding, which is actually a function defined as the opposite of the entropy [51].

We aim to derive the level and the structure of interactions between disciplinary research productivities. We can think of this as an inverse problem, as inference of the underlying network is drawn from observational data [25]. Importantly, Georgesçu-Roegen [22] provides the theoretical support for the unknown model parameters, and justification of the assumptions underlying our statistical model.

### 4.2. Maximum Entropy Estimates

We define our variables as the vectors $s_{i}=\left(s_{i}^{\left(1\right)},\ldots ,s_{i}^{\left(\gamma \right)},\ldots ,s_{i}^{\left(D\right)}\right)$, i=1,…,N. Here and in the following bold style marks a vector quantity. Subscripts, e.g., *i*, indicate disciplines, whereas the superscript index γ=1,…,D refers to a given Country. The observable $s_{i}$ depends on the observation time *t*, thus the set of data $\left\{s\left(t\right)\right\}=\left\{s_{1}\left(t\right),\ldots s_{i}\left(t\right),\ldots ,s_{N}\left(t\right)\right\}$ can be defined, where t=1,…,T identifies a given realization of the generic set of variables or configuration {s}. To simplify the notation, where unnecessary, the index *t* will be omitted. The element of our variable, $s_{i}^{\left(\gamma \right)}$, in the case of our application to knowledge production (see Section 5.1), is related to productivity in the discipline *i* of the Country γ as follows

(2) $$\begin{array}{ccc} s_{i}^{\left(\gamma \right)}\left(t\right) & = & \frac{\Delta_{i}^{\left(\gamma \right)}\left(t\right)}{\sqrt{\sum_{\gamma =1}^{D}\Delta_{i}^{\left(\gamma \right)}{\left(t\right)}^{2}}};\;\;\Delta_{i}^{\left(\gamma \right)}\left(t\right)=\pi_{i}^{\left(\gamma \right)}\left(t\right)-{\bar{\pi }}_{i}\left(t\right); \end{array}$$

(3) $$\begin{array}{ccc} {\bar{\pi }}_{i}\left(t\right) & = & \frac{1}{D}\sum_{\gamma =1}^{D}\pi_{i}^{\left(\gamma \right)}\left(t\right);\;\;\gamma =1,\ldots ,D;i=1,\ldots ,N;\;t=1,\ldots ,T. \end{array}$$

$\pi_{i}^{\left(\gamma \right)}\left(t\right)$ is the productivity of country γ in disciplinary subject category *i* at time *t*. We let ${\bar{\pi }}_{i}\left(t\right)$ represent the world-country average of productivity in subject *i*, so that $\bar{s_{i}}=0$ and $\bar{s_{i}^{2}}=\frac{1}{N}$. We can use this formulation to account for the recent trend of increasing worldwide scientific productivity, considering deviations from the world-country average productivity, $\Delta_{i}^{\left(\gamma \right)}\left(t\right)$, in place of $\pi_{i}^{\left(\gamma \right)}\left(t\right)$. While the average scientific productivity increases over time, the distribution of the deviations around the means does not. See the evidence reported in [52].

Shannon’s [51] theorem states that entropy (*S*), defined in statistical mechanics, is a measure of the ‘amount of uncertainty’ related to a given discrete probability distribution p(s). Accordingly, S[p] is given by

(4) $$\begin{array}{c} S\left[p\right]=-K\sum_{\left\{s\right\}}p\left(s\right)\log\left[p\left(\left\{s\right\}\right)\right], \end{array}$$

where *K* is a positive constant and p({s}) is the pdf (probability density function) of the configuration {s}. This quantity is positive, additive for independent sources of uncertainty and it agrees with the intuitive notion that a uniform (or broad) distribution represents more uncertainty than does a sharply peaked distribution. It is immediate to verify the latter observation in the one-dimensional case by considering Equation (4) and taking into account the property of the discrete distribution of probability, $p_{i}\leq 1$.

In making inference on the basis of incomplete information we must use that probability which maximizes the ‘amount of uncertainty’ or entropy subject to whatever is known [45]. This yields an unbiased assignment, avoiding arbitrary assumption of information which by hypothesis we do not have [45]. For a set of variables {s} the so-called empirical expected value of a given function of {s} is defined as the average of the function over the observed realization (the mean) of {s}, in the present case the average over time. Since some empirical expectation values can be measured, formally this means that p({s}) can be found as the solution of a constrained optimization problem, i.e., maximizing the entropy of the distribution subject to conditions that enforce the expected values to coincide with the empirical ones. We will refer to the quantities whose averages are constrained as ‘features’ of the system. For simplicity, we choose to observe the lower order statistics of the data which can bring information about the underlying network of interactions between variables, i.e., pairwise correlations. The features of the system we are considering are thus the two-variable combinations $s_{i}\cdot s_{j}$, i,j=1,…,N, i≠j. The optimization problem reduces to

(5) $$\begin{array}{c} Max_{p(\{s\})}S\left[p\right], \end{array}$$

with the constraints

(6) $$\begin{array}{cc} & \sum_{\left\{s\right\}}p\left(\left\{s\right\}\right)=1\;\mathrm{and}\;<s_{i}\cdot s_{j}>=\frac{1}{T}\sum_{t=1}^{T}s_{i}\left(t\right)\cdot s_{j}\left(t\right),\;\;i,j=1,\ldots ,N,i\neq j, \end{array}$$

where ‘<>’ is the true average over the distribution *p*. The symbol ‘·’ denotes the Hadamard product of two vectors. The first constraint accounts for the correct normalization of the pdf, whereas the second one arises from the required equality between true and empirical average of the above-defined features. Generally, if certain interdependencies are known to exist between the elements of the matrix S[p], constraints can be imposed to account for these interdependencies. However, instead of imposing a priori interdependency constraints we chose to infer them instead of assuming their existence. Solving Equation (5) with the constraints (6) leads to

(7) $$\begin{array}{c} p\left(\left\{s\right\}\right)=\frac{e^{-\frac{1}{2}\sum_{i\neq j}J_{ij}s_{i}\cdot s_{j}}}{Z}. \end{array}$$

The chosen parameters, $J_{ij}$, are symmetric, i.e., $J_{ij}=J_{ji}$ (There is a link between the assumption of equilibrium underlying a Boltzmann-Gibbs distribution, and symmetry of the pairwise interactions. Symmetric couplings lead to a steady state described by the Boltzmann-Gibbs distribution while asymmetric couplings lead to a non-equilibrium state [53]. We can assign to the system a particular dynamics, which leads it to a given steady state distribution. Recent developments achieved for dynamical inverse Ising model [54,55] could represent an interesting extension of the present work, which is left for future research). The constant *Z* can be determined by exploiting the constraint $\sum_{\left\{s\right\}}p\left(\left\{s\right\}\right)=1$, obtaining $Z=\sum_{\left\{s\right\}}e^{-\sum_{i\neq j}J_{ij}s_{i}\cdot s_{j}}$. The parameters $J_{ij}$ are determined by requiring the second constraint to be fulfilled. Asymmetric $J_{ij}$ can always be re-conducted to symmetric $J_{ij}$, which give rise to the same values of pdf if this latter is the Gibbs distribution in Equation (7).

We observe that the pdf defined in Equation (7) coincides with the Maxwell-Boltzmann probability distribution function at a given fixed temperature,

(8) $$\begin{array}{c} p\left(\left\{s\right\}\right)=\frac{e^{-H(\{s\})}}{Z} \end{array}$$

related to an Ising model with spin $s_{i}$, interaction parameters $J_{ij}$ and zero magnetic field, described by the Hamiltonian

(9) $$H=-\frac{1}{2}\sum_{i\neq j}^{N}J_{i,j}s_{i}\cdot s_{j}.$$

The quantity *Z* in Equations (7) and (8), constant with respect to {s} but dependent on the set of parameters {J} with generic element $J_{ij}$, is called in this context the partition function.

The connection between maximum entropy and maximum likelihood is indeed well known (see e.g., [56]) and the pdf in Equation (7) which satisfies the constraint on the parameters $J_{ij}$ given in Equation (6) can properly be derived by searching the maximum of the so-called Likelihood function within the class of models of the Boltzmann distribution related to an Ising model with zero magnetic field. The Likelihood function is defined in the context of Bayesian inference (see e.g., [26]). By assuming that (i) each realization of the set {s} is drawn independently, (ii) the data have been generated by a (known) model, which depends on the set of (unknown) pairwise parameters {J}, one aims to find the optimal values of {J}, i.e., the ones which maximize the conditional probability p({J}|{s}). From the Bayes theorem [26] it follows that

(10) $$\begin{array}{cc} & p\left(\left\{J\right\}|\left\{s\right\}\right)=\frac{p(\{s\}|\{J\})p(\{J\})}{p(\{s\})}=\frac{p(\{s\}|\{J\})p(\{J\})}{\int_{\left\{J\right\}}p\left(\left\{s\right\}|\left\{J\right\}\right)p\left(\left\{J\right\}\right)}.\;\; \end{array}$$

The probability p({J}|{s}) is called *posterior*, p({J})*prior*, p({s})*evidence* and p({s}|{J}) *Likelihood*. If the prior is the uniform distribution, as we assume here, the most probable a posteriori set of variables is, as a consequence of Equation (10), the one which maximizes the Likelihood function. Under the further assumption that the Likelihood function belongs to the class of Boltzmann distribution functions, the so-called Log-Likelihood function can be defined as

(11) $$\begin{array}{c} l\left(\left\{J\right\}\right)=\sum_{t=1}^{T}\log\left[p\left(\left\{s\left(t\right)\right\}|\left\{J\right\}\right)\right]=\sum_{t=1}^{T}-H\left(\left\{s\left(t\right)\right\}|\left\{J\right\}\right)-T\log\left(Z\left(\left\{J\right\}\right)\right). \end{array}$$

If one assumes that the system can be described by an Ising-like, pairwise interacting model (see Appendix A for an introduction and additional details) with zero external field, the Hamiltonian *H* is the one defined in Equation (9). We observe that in the definition of the Log-Likelihood function in Equation (11) the hypothesis of independency of the realizations of the configuration {s} at different times has been exploited, see point (i) above. Thus, the optimization problem reduces to choosing the set of parameters {J}, which maximize the pdf in Equation (11). A quick calculation of the first and second derivatives of Equation (11) with respect to the parameters $J_{ij}$ shows that the set of parameters which maximizes Equation (11) should indeed maximize Equation (5) with the constraint in Equation (6).

The Ising model has been widely applied in different fields, such as modelling the behaviour of magnets in statistical physics [57], image processing and spatial statistics [58,59,60], modelling of neural networks [61] and social networks [62]. It is, however, worth noting that by exploiting Shannon’s theorem the Ising model does not arise from specific hypotheses about the underlying network but instead is the least-structured model consistent with the measured pairwise correlations. In Appendix A, we outline how the Ising spin glass model is introduced in the physics of complex systems. Table 1 describes the main components of our model and the correspondence between the Ising spin model from statistical physics and economic productivity analysis.

The $J_{ij}$ in physics measure a direct and reciprocal (mutual) effect (the interaction) of one entity on another entity (and vice versa). The concept of interaction in physics can find its correspondence in the interdependency in Input-Output economic analysis. The latter means the existence of a mutual influence between sectors (disciplines).

The coupling parameters $J_{ij}$ generate the configurations of the system that may be characterized by the correlations between the spin variables, the so-called overlap measures (see also [63]), defined as follows:

(12) $$Q_{ij}=1/T\sum_{t=1}^{T}s_{i}\left(t\right)\cdot s_{j}\left(t\right),$$

where t=1,…,T is time. As it is well known, a correlation measures the association between two variables. It shows a tendency of one variable to change with some regularity when the other changes, but this tendency may be moderated (influenced) by other factors, and depends on the whole configuration, including indirect effects. Correlation does not mean a direct effect or relation. On the other hand, interactions or interdependencies ($J_{ij}$) refer to strict relationships between variables which allow us to describe the impact of the variation of one variable on another. Assuming this model to make inference permits us to consider correlations beyond the interdependencies among the units of analysis. As we will see in the application (see Section 5), the productivity of two disciplines may be correlated because they tend to be associated in their variation, but they may not interact. Here we impose $J_{ij}\geq 0$ without loss of generality. The $J_{ij}$ represent the interaction strength between *i* and *j*. The higher the value of $J_{ij}$ the stronger is the interaction between *i* and *j*.

### 4.3. Maximum-Likelihood and Pseudo-Likelihood Estimates

Though the likelihood function definition has deep roots in information theory, Bayesian inference and statistical mechanics, as discussed in the Section above, the realization of an optimization algorithm able to draw the optimal {J} is hindered by the general intractability of computing Z({J}) and its gradient [18,26]. Hence, in place of maximizing the likelihood function, we may define and maximize a different objective function, the so-called pseudo-likelihood. It is possible to show [18,64] that the estimation of the parameters obtained by a pseudo-likelihood maximization is consistent with the maximization of the likelihood function, that is the two functions are maximized by the same set of parameters. This statement becomes exact in the case of infinite sampling [18]. We do not discuss in detail here how these results are achieved. We discuss only the guidelines, redirecting for details elsewhere (see e.g., [65] and the references cited in this sub-section). By its very establishment, the pseudo-likelihood function permits to solve the optimization problem avoiding the troubles related to the computation of Z({J}). It has indeed the advantage to be maximized in polynomial time. The pseudo-likelihood function is based on the so-called local conditional likelihood functions, $p(s_{i}|\left\{s_{\setminus i}\right\})$ at each node of the network, $s_{i}$, i=1,…,N. The symbol $\left\{s_{\setminus i}\right\}$ means a set of variables $s_{j}$ with i≠j. The local conditional probability (single variable pseudo-likelihood) at the *i*-th node is

(13) $$\begin{array}{c} p\left(s_{i}|\left\{s_{\setminus i}\right\}\right)=\frac{1}{Z_{i}}e^{-H_{i}\left(s_{i}|\left\{s_{\setminus i}\right\}\right)} \end{array}$$

The local Hamiltonian $H_{i}\left(s_{i}|\left\{s_{\setminus i}\right\}\right)=-s_{i}\cdot \left[\frac{1}{2}\sum_{i\neq j}^{1,N}J_{i,j}s_{j}\right]$ and the local partition function is $Z_{i}=\sum_{\left\{s_{i}\right\}}e^{-H_{i}\left(s_{i}|\left\{s_{\setminus i}\right\}\right)}$. Letting $l^{\prime }\left(s_{i}|\left\{s_{\setminus i}\right\}\right)=\log\left[p\left(s_{i}|\left\{s_{\setminus i}\right\}\right)\right]$, the Log-Pseudo-Likelihood function is

(14) $$\begin{array}{c} \lambda \left(\left\{J\right\}\right)=\sum_{t=1}^{T}\sum_{i=1}^{N}l^{\prime }\left(s_{i}\left(t\right)|\left\{s_{\setminus i}\left(t\right)\right\}\right). \end{array}$$

The gradient of the log-pseudo-likelihood function with respect to the parameter $J_{ij}$ can be easily calculated, obtaining

(15) $$\begin{array}{c} \frac{\partial }{\partial J_{ij}}\lambda \left(\left\{J\right\}\right)=\frac{1}{2}T[\frac{1}{T}\sum_{t=1}^{T}s_{i}\left(t\right)\cdot s_{j}\left(t\right)-<s_{i}\cdot s_{j}{>}_{i,\{J\}}], \end{array}$$

where ‘$<\;\;{>}_{i,\{J\}}$’ states for ensemble average calculated for the pdf $p(s_{i}|\left\{s_{\setminus i}\right\})$ with parameters set {J}. It is possible to rephrase it, obtaining

(16) $$\begin{array}{c} \frac{\partial }{\partial J_{ij}}\lambda \left(\left\{J\right\}\right)=\frac{1}{2}T[\frac{1}{T}\sum_{t=1}^{T}s_{i}\left(t\right)\cdot s_{j}\left(t\right)-<<s_{i}\cdot s_{j}{>}_{i,\{J\}}{>}_{\left\{J\right\}}], \end{array}$$

where ‘$<\;{>}_{\left\{J\right\}}$’ states for ensemble average calculated with the pdf in Equation (7) or Equation (8) with set of parameters {J}. The gradient of the log likelihood function is

(17) $$\begin{array}{c} \frac{\partial }{\partial J_{ij}}l\left(\left\{J\right\}\right)=\frac{1}{2}T[\frac{1}{T}\sum_{t=1}^{T}s_{i}\left(t\right)\cdot s_{j}\left(t\right)-<s_{i}\cdot s_{j}{>}_{\left\{J\right\}}] \end{array}$$

By comparing Equations (15) and (16) it is possible to infer that in the limit of large *T*: (i) both the gradients go to zero if the elements of the set {J} are the ‘true’ parameters defining the pdf which generates the data; (ii) $\frac{\partial }{\partial J_{ij}}\lambda \left(\left\{J\right\}\right)\to \frac{\partial }{\partial J_{ij}}l\left(\left\{J\right\}\right)$.

Specifics on computation of log-pseudo-likelihood function and its gradient with respect to $J_{ij}$, and details on optimization algorithm are reported in Appendix B.

The methods employed and the codes written to implement the related algorithms have been tested on the Ising model with known coupling coefficients on random graphs, thus guaranteeing the right convergence of the inference procedure and the proper reconstruction of the interaction network.

## 5. Application to Knowledge Production

### 5.1. The Knowledge Production Network

**Figure 3:**
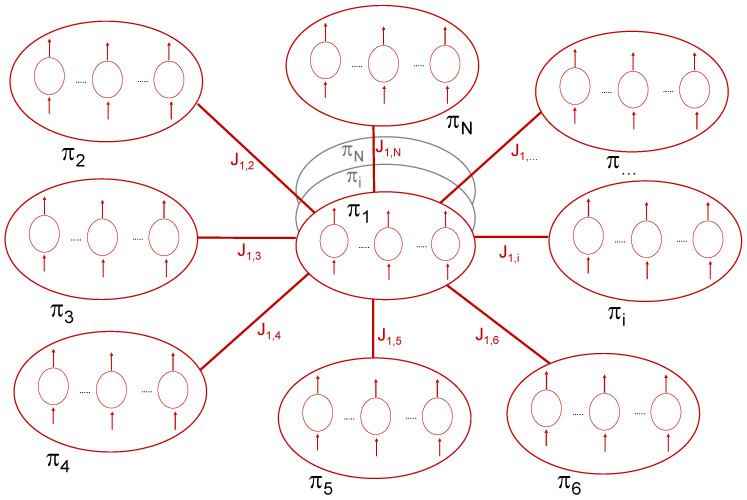
An illustration of the network model. Each disciplinary productivity $\pi_{i}$, which is a node in this network, includes the country-level set of disciplinary productivity $\pi_{i}^{\gamma }$ with γ=1,…,D.

We point out that our proposed inferential approach used to recover the broader network structure can be applied to different network models developed for diverse fields of applications. To illustrate its potential, we apply the method to the field of knowledge production. Taking into account the data available for the empirical analysis, we will work with a network that has the form shown in Figure 3.

In the context of the GRFF model we assume that accumulated knowledge from previous periods is a fund variable and new knowledge produced in the form of publications in the current period is a flow. Two fund sources arise from accumulated knowledge: knowledge that a DMU itself produced in previous periods, z, and spillover knowledge accruing from the knowledge (publications) that other DMUs produced in previous periods, Y. In turn, the flow of new knowledge produced by a DMU in the current period becomes part of the fund of own accumulated knowledge it can draw upon in a subsequent period and that new knowledge spills over and becomes a fund available to other DMUs in subsequent periods.

Figure 3 illustrates the structure of the network that we will reconstruct using our new semi parametric approach in Section 5. This network models the interdependencies existing between disciplinary productivity/efficiency, $\pi_{i}$, where disciplines i=1,…,N are the Scopus subject categories. The network nodes $\pi_{i}$ comprise the respective productivity measures, $\pi_{i}^{1},\ldots ,\pi_{i}^{\gamma },\ldots ,\pi_{i}^{D}$, for each country γ=1,…,D. We consider the main 53 world countries according to their scientific production. The productivity/efficiency of country γ in discipline *i*, $\pi_{i}^{\gamma }$ (omitting the time *t* from the notation for an easier reading), is computed in a DEA setup through the Shephard output distance function for each country relative to the discipline-time specific technology ($P_{i}^{\gamma ,t}$ that will be introduced in detail in Section 5) as

(18) $$\pi_{i}^{\gamma }\left(x_{i}^{\gamma ,t},z_{i}^{\gamma ,t},Y_{i}^{\gamma ,t},y_{i}^{\gamma ,t}\right)=\inf\left\{\pi_{i}^{\gamma }:y_{i}^{\gamma ,t}/\pi_{i}^{\gamma }\in P_{i}^{\gamma ,t}\right\}.$$

The reciprocal of the Shephard output distance function, $1/\pi_{i}^{\gamma }$, measures the proportional expansion of observed outputs that could be achieved if the DMU were to become efficient.

Figure 3 shows a network of disciplinary productivities $\pi_{i}$, each of them composed by country-level productivity $\pi_{i}^{1},\ldots ,\pi_{i}^{\gamma },\ldots ,\pi_{i}^{D}$. For instance, the productivity of Chemistry $\pi_{Chem}$ is composed by the country-level productivity $\pi_{Chem}^{Arg},\ldots ,\pi_{Chem}^{\gamma },\ldots ,\pi_{Chem}^{USA}$, where ‘Arg’ stands for Argentina.

The disciplinary interdependencies or interactions are represented by the pathways, $J_{ij}$. Importantly, these pathways are generally unknown, and must be inferred, although if knowledge of the interactions is known, constraints can be incorporated that account for those interactions. Example disciplinary interdependencies include the use of new computational methods from computer science by those in mathematics or the natural sciences; advances in neuroscience by those in medicine and nursing; new findings in environmental science by those in earth sciences and agriculture. Indeed, as we will see in the application, the present study illustrates an interdependency pathway between physics and the social sciences. Although it is theoretically possible to include the inputs/outputs of all disciplines in the technology, by doing this we would encounter two problems. First, assuming the homogeneity of all disciplines in their knowledge production, which is clearly not the case, and second, the curse of dimensionality would likely render all DMUs to be on the efficient frontier. Therefore, we first estimate productivity/efficiency for specific disciplines within a specific country in an NDEA model. Then, we use those productivity/efficiency estimates to infer the generally unknown connections between disciplines using the method described in Section 4.2.

### 5.2. Data and Descriptive Analysis

Our world-country bibliometric data were extracted from the Scopus database, for 16 disciplinary subject categories from 1996 to 2012. Data problems in bibliometric studies are well known. A common way to reduce them is to analyze macro-level bibliometric data. Comparative analysis is more reliable when the unit of analysis is more aggregated because in a larger sample size, micro random errors mutually compensate [66]. The potential for changes in coverage from inclusion or exclusion of journals to disproportionately affect smaller countries with fewer publications presents another issue of concern. This may lead to unreliable values when a country only has a small number of scholarly outputs [67]. To avoid this problem, we consider the 53 most productive countries (in terms of scientific productivity), which account for more than 95% of the world scientific production in the considered period. (The 53 countries (Country Alpha 3 Code labels) analysed in this paper are: 1 = ARG, 2 = AUS, 3 = AUT, 4 = BEL, 5 = BGR, 6 = BRA, 7 = CAN, 8 = CHE, 9 = CHL, 10 = CHN, 11 = COL, 12 = CZE, 13 = DEU, 14 = DNK, 15 = EGY, 16 = ESP, 17 = FIN, 18 = FRA, 19 = GBR, 20 = GRC, 21 = HKG, 22 = HRV, 23 = HUN, 24 = IND, 25 = IRL, 26 = IRN, 27 = ISR, 28 = ITA, 29 = JPN, 30 = KOR, 31 = MEX, 32 = MYS, 33 = NGA, 34 = NLD, 35 = NOR, 36 = NZL, 37 = PAK, 38 = POL, 39 = PRT, 40 = ROU, 41 = RUS, 42 = SAU, 43 = SGP, 44 = SRB, 45 = SVK, 46 = SVN, 47 = SWE, 48 = THA, 49 = TUN, 50 = TUR, 51 = TWN, 52 = UKR, 53 = USA).

Luwel [68] and Aksnes et al. [69] report the main issues related to the integration of bibliometric data with other inputs data, in particular R&D expenditures. Methodological problems in measuring productivity at the macro level are mainly due to a lack of standardization in the measurement of resources and outcomes across countries. Moreover, the methodologies for collecting input and output data have been developed largely independently from each other. We attempt to bypass these issues of standardization and measurement by working with simpler quantity data on inputs and outputs for this paper. We restrict research inputs to the number of publishing authors, NA, and outputs to the number of publications, *P*, as well as the number of highly cited publications (top 10%), HCP. These indicators are the most known and commonly used indicators for the assessment of the contributions scholars make in their research publications to the advancement of scholarly knowledge [70]. Bibliometrics and quantitative studies of science are heavily related to these indicators [71]. The data on NA come from Elsevier Bibliometric Research Project (EBRP). In the elaborations carried out to estimate the interactions ($J_{ij}$) and infer the network structure, to increase the number of available data we transformed yearly data into weekly data by means of a linear interpolation. This leaves 833 observations of weekly total publications and highly cited publications for the 1996–2012 panel.

Table 2 gives an overview of all the indicators available for the present study. The list of the Scopus 27 subject categories is reported in Table 3.

Table 3 reports in bold the 16 subject categories considered in the analysis and the variables available for each discipline. We excluded social sciences and humanities disciplines whose coverage of scholarly outputs in indexed journals is much lower than the other subject categories considered. Table 4 presents descriptive statistics for the selected disciplines, namely: BIOC, COMP, ENGI, MEDI and PHYS. Appendix C reports descriptive statistics on the other disciplines that will be analysed in the paper.

### 5.3. Models for Estimating Knowledge Production

Our network models borrow from previous work on knowledge production ([6,30]), while the production variable choices are modeled after Georgesçu-Roegen, where we include both flow variables and funds variables. We think of knowledge production in a production axiomatic framework, in which we include author count as a flow type input variable, and cumulated previous own publications as a fund or knowledge stock, which produces a flow of publication outputs.

What makes it a network is the fact that the cumulated publications of a discipline in one country are available to other countries in the same discipline, and previous cumulated publications from other countries in that discipline also are available to the discipline in the ‘home’ country as a fund-type input variable. This specification proxies the public good/externality nature of publications as well as their role in contributing to the stock of knowledge.

Table 5 outlines the examined basic (static) and network productivity models, which we separate into two categories: Quantity (1) and Quality (2). The first category represents ‘quantity of knowledge,’ using raw publication counts (*P*) to measure final knowledge output. Within this category, the basic model (1.1) uses the stock of previous publications within each country and the number of authors (NA) as the knowledge inputs. The corresponding network model (1.2) also includes the stock of previous publications from other countries as a knowledge spillover input. The second category represents ‘quality of knowledge,’ using the number of highly cited publications (HCP) in place of raw publication counts, for both previous publication inputs and current publication final outputs. While citations are widely used to indicate publication quality, we note that there are several other potential alternatives, including SCImago journal rank (SJR) (Lee et al., 2016), peer reviews [72], and restricting to top-tier journals [73]. Quality models 2.1 and 2.2 serve as the quality-adjusted versions of quantity models 1.1 and 1.2, using the HCP approach to distinguish quality. We add a simple productivity model given by the ratio of publications to authors (P/NA) as a baseline.

**Figure 4:**
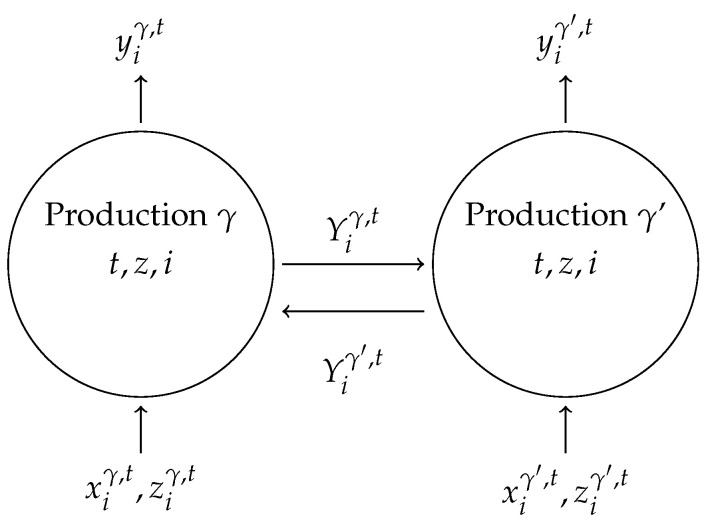
Network Technology for Knowledge Production.

Figure 4 illustrates the network for the hypothetical case of two countries, γ and $\gamma^{\prime }$, both for discipline *i* in period *t*. Their flow inputs are the author counts denoted by *x* and their final outputs are denoted by *y*. The previous publication fund variables that provide the network connection are denoted as *Y* and the own previous fund variables are denoted by *z*.

### 5.4. Production Efficiency Estimated Using NDEA

To fix ideas, and following [6], let each country be indexed by γ=1,…,D, for periods t=1,…,T. We will augment their model by including disciplines i=1,…,N, here N=16. Denote flow input as $x^{\gamma ,t}$ (here a scalar, but possibly a vector). Fund variables include own country cumulated past publications denoted as $z_{i}^{\gamma ,t}$, and other country cumulated previous publications as $Y_{i}^{\gamma ,t}$. Final output is own country current period publications denoted as $y_{i}^{\gamma ,t}$.

Again, following [6], we define the own country fund variable *z* as the sum of the previous 3 periods’ publications for that country, which we denote as $z_{i}^{\gamma ,t}=\sum_{\tau =1}^{3}y_{i}^{\gamma ,t-\tau }$, where τ=1,2,3 represents the 3 previous years. Similarly, we use $Y_{i}^{\gamma ,t}=\sum_{\tau =1}^{3}\sum_{\gamma^{\prime }\neq \gamma }^{D}y^{\gamma^{\prime },t-\tau }$ to represent spillover knowledge from other countries’ previous publications.

We can now state the formal problem we are estimating to solve for efficiency in the NDEA model. As explained in Section 5.1, we employ the Shephard output distance function as a performance measure for each country, i.e., it is the explicit objective function. The distance function for country γ in discipline *i*, $\pi_{i}^{\gamma }$, is defined as

(19) $$\pi_{i}^{\gamma }\left(x_{i}^{\gamma ,t},z_{i}^{\gamma ,t},Y_{i}^{\gamma ,t},y_{i}^{\gamma ,t}\right)=\inf\left\{\pi_{i}^{\gamma }:y_{i}^{\gamma ,t}/\pi_{i}^{\gamma }\in P_{i}^{\gamma ,t}\left(x_{i}^{\gamma ,t},z_{i}^{\gamma ,t},Y_{i}^{\gamma ,t}\right)\right\}.$$

This objective function scales observed country output to the frontier of the output set $P^{\gamma ,t}\left(.\right)$ and takes a value of unity for a country on the frontier and a value less than one for a country that is below the frontier. Using DEA (see Section 3.1), the reference technology (output set) for period *t*, serves as the constraints in our problem:

$P^{t}\left(x_{i}^{\gamma ,t},z_{i}^{\gamma ,t},Y_{i}^{\gamma ,t}\right)=\left\{y:\left(x_{i}^{\gamma ,t},z_{i}^{\gamma ,t},Y_{i}^{\gamma ,t}\right)\mathrm{can}\mathrm{produce}y\right\}$ may be written

(20)Piγ,t(xiγ,t,ziγ,t,Yiγ,t)={yt:∑γ=1Dλiγ,tyiγ,t≧y,(21)∑γ=1Dλiγ,txiγ,txiγ,t,(22)∑γ=1Dλiγ,tziγ,t≦ziγ,t(23)∑γ=1Dλiγ,tYiγ,t≦Yiγ,t,λiγ,t≧0,γ=1,…,D;i=1,…,N;t=1,…,T,}

where the $\lambda_{i}^{\gamma ,t}$ are intensity variables that form the best-practice frontier technology from the observed inputs and outputs. Equation (20) is the constraint with respect to own country current output (publications), Equation (21) is the input flow constraint, Equation (22) is a fund constraint with respect to own cumulated previous publications and Equation (23) is a fund constraint with respect to other country cumulated publications–the spillover constraint. We solve this problem for each discipline in each country using linear programming. These solutions are the data for the second stage which estimates the correlations Q and the parameters J, as described below.

As described in Section 4.2, the statistical analysis of the knowledge production we propose here is developed in the framework of information theory, where through the well-known Shannon theorem, the entropy is introduced and defined. We can measure the so-called pairwise correlation functions or overlaps from the collected data. Assuming that we only get this information, by following the line of the Shannon theorem it is possible to find the class of the probability distribution (which will depend on some adjustable parameters, the set J), which maximizes the entropy with the constraint that the pairwise correlations obtained from such probability distribution match the measured ones. In this way the probability distribution (see Equation (12) with the Hamiltonian of the so-called Ising model (see Equation (13)), and also Appendix A), is directly obtained by maximizing the entropy introduced in the Shannon theorem with the aforementioned constraint, without any further unverifiable (thus arbitrary) hypothesis. In this respect, the entropy introduced in the Shannon theorem represents a measure of the ‘amount of uncertainty’. For example, limiting to the case of a discrete probability distribution, if any constraint is superimposed (i.e., any a priori knowledge is available), the entropy function introduced by Shannon is maximum for a uniform distribution. This agrees with our intuitive notion that a broad distribution represents more uncertainty than a sharply peaked one. Figure 3 is a sketch of an Ising model whose variables are defined in the knowledge production framework. It is thus linked to the entropy defined from information theory because the adoption of the Ising model is a direct consequence of the maximum entropy principle, as discussed above.

### 5.5. Results

We estimate our two models for each of the 53 countries and 16 disciplines, reported in Table 3. To summarize the results, we present the annual output share-weighted geometric means of the productivity values by discipline, for the alternate versions of Models 1 and 2: Total publications (basic and network, 1.1 and 1.2) and highly cited publications (basic and network, 2.1 and 2.2). For comparison with our model estimates we include the simple ratio of publications to authors (P/NA). The rank order correlations of P/NA and estimated production efficiency are reported in Table 6 for selected disciplines: Computer Science (COMP), Engineering (ENGI), Medicine (MEDI) and Physics (PHYS). Not surprisingly, the lowest correlations occur for the basic DEA quantity model (1.1) and the NDEA quality model (2.2). In contrast, the two quantity models (1.1 and 1.2) and the two quality models (2.1 and 2.2) tend to have the highest correlations in each of the disciplines.

We are interested in studying the interdependencies across disciplines as well as within disciplines at a macro or country level. The interactions between knowledge production efficiency may differ by type and magnitude, as well as their ultimate effects on the associated disciplines and countries. Our NDEA estimates provide within disciplinary connection across countries, but not across disciplines.

For instance, collaboration between researchers in two disciplines, say physics and health can mean that gains in efficiency in one discipline are reinforced by gains in efficiency in another discipline. Interdisciplinary research requires that the researchers in each discipline to learn the vocabulary, terminology, the notation, the ideas, and so forth of other disciplines. Those disciplines and researchers who can overcome the transaction costs of learning the vocabulary, terminology, etc. can expand this more general/interdisciplinary knowledge. Our method identifies those disciplines where such gains are possible. In contrast, if interdisciplinary transaction costs are too high, as might occur when researchers come from two very different disciplines, then gains in efficiency in one discipline may serve to lower efficiency in another discipline.

There may also be cases in which two or more disciplines with different levels of production efficiency interact such that the more productive disciplines slow down while the less productive disciplines increase their productivity. For this reason, analyzing the correlations or indirect connections (the overlap measures $Q_{ij}$ introduced in Equation (12)) between (disciplinary) efficiency levels can be interesting. However, going further and estimating their interdependencies ($J_{ij}$) can provide more useful information to analyze the way in which scientific knowledge is produced and organized worldwide. This information could be useful for policy makers who determine which disciplines or topics to prioritize and how to distribute research funds among disciplines.

**Figure 5:**
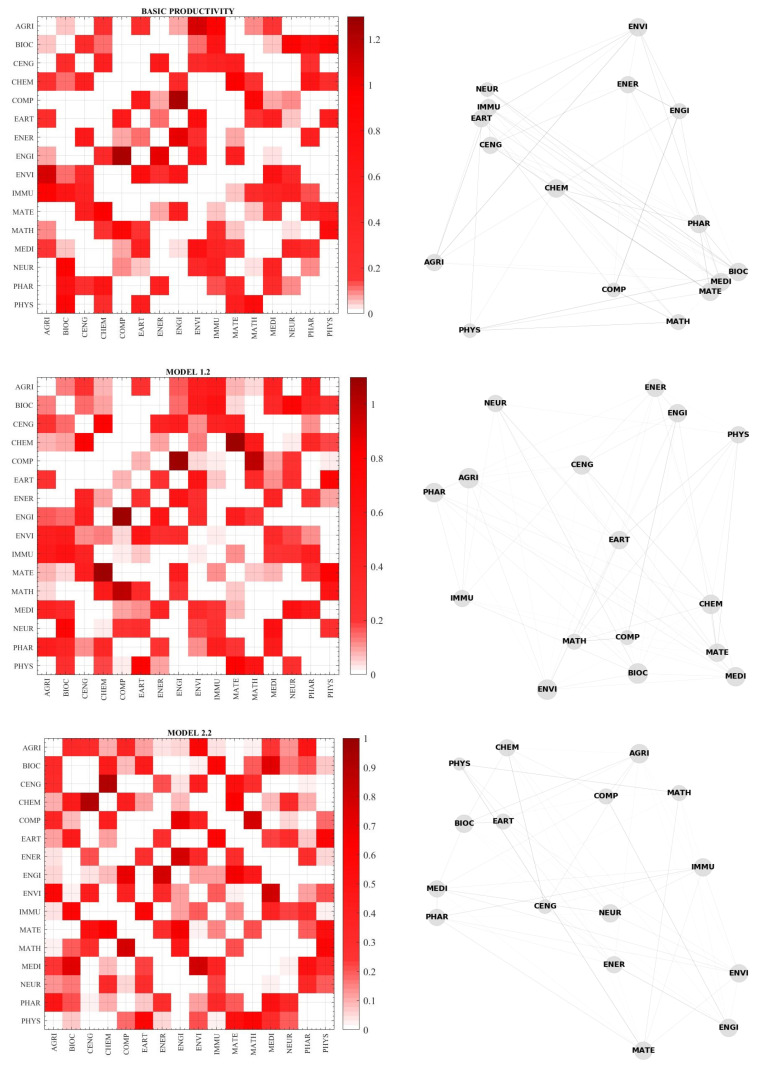
Estimated $J_{ij}$ (**left panels**) and inferred networks (**right panels**) for the three production efficiency models. Top panels refer to the simple productivity model (P/NA), middle panels show the network quantity model 1.2 and the bottom panels show the network quality model 2.2 results.

Figure 5 shows the estimated interdependency parameters ($J_{ij}$) -left panels- and the inferred networks -right panels for the three production efficiency models. Top panels refer to the simple productivity model (P/NA), middle panels show the Model 1.2 (network quantity model) and the bottom panels report Model 2.2 (network quality model) results. In the left panels of Figure 5, the darker squares indicate higher $J_{ij}$ and the NW to SE diagonal comprises all white squares since $J_{ii}=0$ by hypothesis.

The reconstructed networks reported in the right panels of Figure 5 are derived from the estimated $J_{ij}$ obtained by the maximization of the pseudo-likelihood function. The $J_{ij}$ are the edges. The diameter of the node for discipline *i* is proportional to the number of interactions $J_{ij}$. The thickness of the edge depends on the intensity of the related interaction.

**Figure 6:**
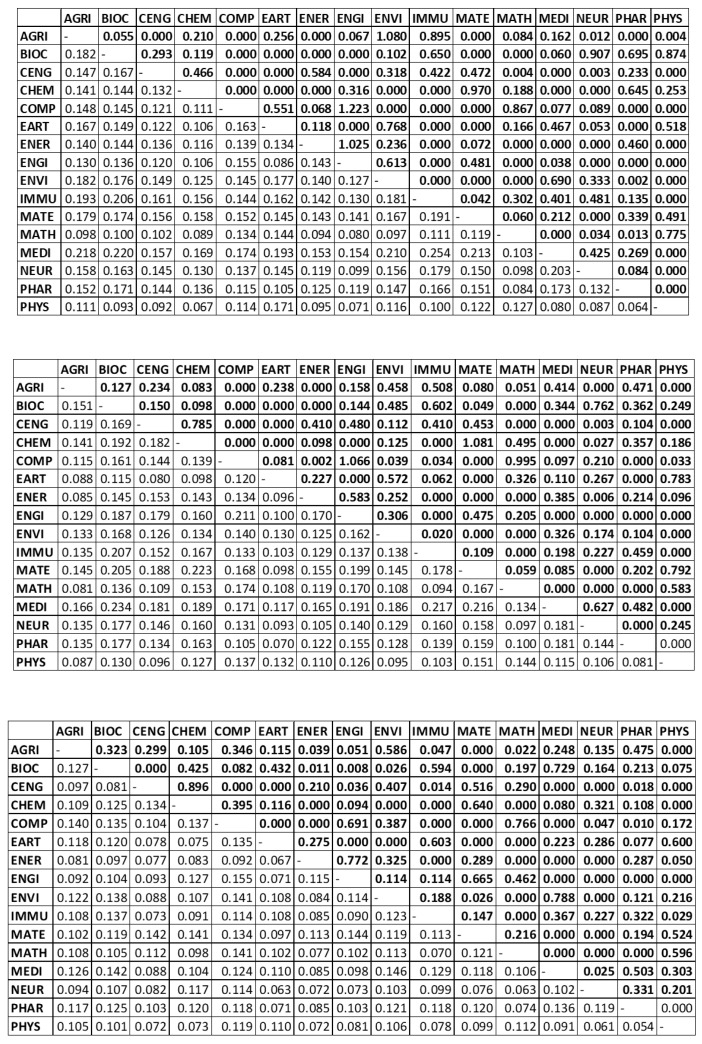
Overlaps ($Q_{ij}$) and Interdependencies ($J_{ij}$) of the simple productivity model (P/NA) (**top panel**), the network quantity model 1.2 (**middle panel**) and the network quality model 2.2 (**bottom panel**). The Northeast values reported in bold are the $J_{ij}$ while the Southwest values correspond to the $Q_{ij}$. See Table 5 for model specifications.

Figure 6 shows the calculated overlap measures ($Q_{ij}$) and the estimated interdependencies ($J_{ij}$) for the different productivity models. The methodology introduced in Section 4 hence allowed us to empirically infer the network structure existing among disciplinary productivity going beyond the simple overlap (or correlations) measures ($Q_{ij}$).

For instance, we can analyze the correlations and interdependencies between the productivity of disciplines CHEM with IMMU and MATE. In the basic quantity model (Figure 6—top panel) CHEM and IMMU and CHEM and MATE show the same overlap measure $Q_{ij}=0.16$, meaning that their production efficiency tend to be positively associated. On the other hand, their respective interdependencies are different. In fact, $J_{ij}$ between CHEM and IMMU is zero, while the interdependency between CHEM and MATE is 0.970, meaning that the productivities of CHEM and MATE present a high level of interdependency (mutual interaction).wae Similarly, the correlations (indirect connection) between PHYS with IMMU and MATE are respectively 0.10 and 0.12, while their interdependencies are respectively 0 and 0.49. This means that PHYS interacts with MATE, but not with IMMU, although the respective production efficiencies are correlated.

Inspecting the middle and the bottom panels of Figure 6 we note that the interaction ($J_{ij}$) between PHYS and MATE is 0.79 in the quantity model (1.2) and 0.52 in the quality model (2.2) while the respective correlations are 0.15 and 0.1.

We can conduct a comparative qualitative analysis between the three different models to explain the results and how the technique works. We solved an indirect problem to estimate the $J_{ij}$ values for each dataset and each model separately, so the inferred network structure is the optimal structure for each dataset.

For instance, the values of $J_{AGRI,PHAR}$ in the three panels of Figure 6 are 0.000 (top), 0.471 (middle) and 0.475 (bottom). These values indicate there is no interdependency between AGRI and PHAR in the simple productivity model (P/NA) as the score is zero, while the interdependencies between AGRI and PHAR are much stronger in the quantity and quality models in which the knowledge production assumes as inputs NA, own previous publications and other previous publications and as output own current publications (Mod. 1.2) while Model 2.2 considers the same inputs/output than model 1.2 but uses the number of Highly Cited Publications (HCP). Similarly, $J_{MATH,PHAR}$ is 0.013 according to the simple productivity model, showing a weak interaction among the two simple productivities estimated by the number of publications per author, while there is no interaction between the productivities of the two disciplines if we measure them according to Model 1.2 and 2.2 (see Table 5) as the J values for MATH and PHAR in middle and bottom panels are zero.

We can also compare the simple productivity model (P/NA) with the quantity models (1.1 and 1.2) to consider any possible effects posed by the curse of dimensionality, given our sample size and model dimensions. The overall results are quite similar: the only differences are due to the DEA modelling and so we should prefer the results of the Model 1.2 which accounts for the Georgesçu-Roegen’s fund’s modelling of the knowledge production.

We then compared the quantity vs. quality results for the network models (1.2 and 2.2), to see whether the obtained estimates were consistent. We found for instance that the interaction between Physics (PHYS) and Computer Science (COMP) in the quantity model (1.2) is 0.033 while their interaction in the quality model (2.2) increases up to 0.172. In contrast, the interaction between Physics and Chemistry that is 0.186 in the quantity model goes down to zero in the quality model.

As we may expect, the interactions between CHEM and CENG are quite high in all the three models (0.466 in the simple productivity model, 0.785 in Mod. 1.2 and 0.896 in Mod 2.2); other expected results are the high interactions of COMP with ENGI and MATH because these disciplines share the same community. A striking result is the interactions we observe between PHYS and MEDI which is quite high in the Quality model (Mod. 2.2, with a value of 0.303) but absent in the Quantity model (Mod. 1.2). A policy implication of this result could be made in the discussion about supporting Societal Challenges that are focused on Medical Sciences and the important role that Physics could play in this context.

The results commented in this section show the usefulness of the analyses carried out in the previous section to shed deeper and new light on the interactions among disciplinary productivity that we would not have been able to derive if we had reduced the analysis to the simple productivity model (P/NA). Differently from existing bibliometric literature [74,75] we consider not only the outputs of scientific production, but also their efficiency in knowledge production. In addition, differently from existing efficiency literature [6] we estimate the interdependencies among disciplinary efficiency thanks to the application of the inferential approach proposed in Section 4.

## 6. Discussion and Conclusions

Network Data Envelopment Analysis (NDEA) models are often used to measure producer performance when separate production technologies are linked across divisions, time, and spillovers can occur between different producers. In theory, NDEA models can accommodate complex, multi-product technologies with inputs used to produce intermediate products and final outputs. However, in practice, the degree of complexity a researcher can introduce is limited by the number of available observations used to construct the technology in what is known as the curse of dimensionality. It is also not obvious how to test for the appropriate structure of the network. In this paper we address these issues with a new theoretical method based on Shannon’s entropy that allows us to infer wider linkages between various producers without having to specify those links within the NDEA model. Our method specifies a NDEA technology and provides nonparametric estimates of producer performance relative to that assumed technology. Then, in a second stage, we employ a semiparametric Bayesian framework that allows us to estimate, rather than assume, the network structure. This second stage exploits advances in the physics of complex systems, machine learning and econometrics of information and reveals additional linkages in the network—in our case—allowing us to infer connections between knowledge disciplines.

While we consider our main contribution to be providing an inferential method to identify structure in NDEA models, we consider our application to knowledge production to be of interest in its own right. The economics of science [76] reminds us that researchers do research for different reasons, including their interest in “puzzle solving”, reputation based on the priority of their discovery, awards and recognition for their achievements, and also through publications which can play a key role in funding and promotion. Research is also a public good, generating knowledge spillovers that can be difficult to capture and quantify. Like any public good, this can lead to underprovision in the market.

The economics of science tells us that the production of scientific research involves multiple inputs, including knowledge, time, materials and equipment. Some inputs are embedded in people (knowledge and time in particular), and most of these inputs are expensive. As observed by Stephan [76], incentives and cost matter for science and economics, particularly for shaping the most efficient mix of resource allocation across disciplines.

In this paper, we combine concepts from economic production theory, Bayesian statistics, and the physics of complex systems to infer the cross-disciplinary and cross-country interactions of research activities. Such understanding is key to achieving the efficient mix of resources for research. The NDEA estimates can be used to derive correlations between disciplines and those same NDEA estimate can be used in a second stage to infer interdependencies between disciplines. For instance, controlling for the quality of publications, math and medicine exhibit positive association, but zero interdependency. In other cases, such as physics and math, there are relatively low correlations of productive efficiencies between the two disciplines, but a high interdependency. We find non-trivial interactions in many cases, which seems promising for future work in this area. Our framework and results could be of particular interest to policy-makers and agencies tasked with prioritizing research funding areas. For instance, the relatively high interaction between physics and medicine might suggest the need to include topics from the physical sciences in new funding for medical research.

This approach could also be applied to other knowledge network systems beyond academic research, such as innovation and technology advance for industrial processes. For instance, a similar framework could be used to estimate the interdependencies of structural industrial profiles, in the form of industrial value added, and structural innovative/technological profiles, based on patents. Inference of the underlying network topology could be used to target research and development funds within the firm, and target public investment decisions.

Summing up more generally, the proposed statistical inferential framework may be applied in a variety of productivity network problems, to infer the underlying structure of the network. The framework developed here is based on complex systems behavior modeling and estimation. There are many possible extensions, left for further studies, including the implementation of out-of-equilibrium time-dependent Ising model.

## Figures and Tables

**Table 1:** Model’s components.

Statistical Physics	Productivity Analysis
Generalized Multicomponent spin model	Disciplinary productivity, defined in
with arbitrary interactions network	DEA in an input-output framework
node variable or multicomponent spin:	deviation from world-country average productivity
$s_{i}=\left(s_{i}^{1},\ldots ,s_{i}^{\left(\gamma \right)},\ldots ,s_{i}^{N}\right)$	of discipline *i* and countries (γ)
Pairwise node *interactions* or *couplings*: $J_{i,j}$	pairwise interdependencies between
	productivity of different disciplines
*Hamiltonian*:	generalized cost function
$H=-\frac{1}{2}\beta \sum_{i,j=1}^{N}J_{ij}s_{i}\left(t\right)\cdot s_{j}\left(t\right)-\sum_{i=1}^{N}s_{i}\left(t\right)\cdot h_{i}$	linked to the estimation of the likelihood
β: inverse of the temperature	external global parameter
$h_{i}$: local magnetic field	contextual environmental variables of discipline *i*

**Table 2:** List of Indicators.

Ind	Description
*P*	Number of articles (integer count)
$P_{f}$	Number of articles (fractional count, based on authors affiliations)
*C*	Total citations (4 years window, i.e., for articles in 2006,
	citations are from 2006–2009)
CPP	Total citations per paper (4 years window, i.e., for articles in 2006,
	citations from 2006–2009)
HCP	Number of articles in top 10 per cent of most highly cited
	articles in a discipline
PINT	Number of internationally co-authored papers
PNAT	Number of nationally (but not internationally) co-authored papers
PINST	Number of papers co-authored by members of different
	institutions within a country
PSA	Number of non-collaborative (single address) papers
NA	Number of publishing authors

**Table 3:** List of the 27 Scopus’ subject categories.

asjc	Subject	Description
Code	Category	
10	GENE	General
11	**AGRI**	Agricultural and Biological Sciences
12	ARTS	Arts and Humanities
13	**BIOC**	Biochemistry, Genetics and Molecular Biology
14	BUSI	Business, Management and Accounting
15	**CENG**	Chemical Engineering
16	**CHEM**	Chemistry
17	**COMP**	Computer Science
18	DECI	Decision Sciences
19	**EART**	Earth and Planetary Sciences
20	ECON	Economics, Econometrics and Finance
21	**ENER**	Energy
22	**ENGI**	Engineering
23	**ENVI**	Environmental Science
24	**IMMU**	Immunology and Microbiology
25	**MATE**	Materials Science
26	**MATH**	Mathematics
27	**MEDI**	Medicine
28	**NEUR**	Neuroscience
29	NURS	Nursing
30	**PHAR**	Pharmacology, Toxicology and Pharmaceutics
31	**PHYS**	Physics and Astronomy
32	PSYC	Psychology
33	SOCI	Social Sciences
34	VETE	Veterinary
35	DENT	Dentistry
36	HEAL	Health Professions

**Table 4:** Summary Statistics Performance Variables, by Discipline (ASJC code) (53 Countries, 1996–2012, 901 Observations Each Discipline).

BIOC (13)	Mean	Std. Dev.	Min	Max
Articles	4357.6	9723.4	44	85,295
Highly Cited	529.1	1572.1	0	15,480
Number of Authors	10,499.4	23,931.7	95	229,139
COMP (17)				
Articles	2727.5	7315.9	7	80,276
Highly Cited	355.6	1013.9	0	10,123
Number of Authors	4161.6	11,548.7	11	128,273
ENGI (22)				
Articles	5433.7	14,078.9	31	156,349
Highly Cited	739.5	1804.5	0	19,830
Number of Authors	8981.8	23,456.8	54	293,605
MEDI (27)				
Articles	7672.4	17,175.3	95	165,181
Highly Cited	1027.9	2929.3	0	28,743
Number of Authors	15,773.2	34,286.3	211	351,702
PHYS (31)				
Articles	4396.5	8271.2	19	58,244
Highly Cited	559.9	1208.5	0	10,591
Number of Authors	6946.0	14,391.0	26	127,209

**Table 5:** Network DEA Models of Knowledge Production.

Models	Inputs	Output
**(0) Simple**		
**productivity model**	Own author count (NA)	Own current pubs (*P*)
**(1) Quantity**		
1.1 (Basic)	NA, own prev pubs	*P*
1.2 (Network)	NA, own prev pubs, other prev pubs	*P*
**(2) Quality**		
2.1 (Basic)	NA, own prev HCP	Own current HCP
2.2 (Network)	NA, own prev HCP, other prev HCP	Own current HCP

**Table 6:** Rank Correlations of Productivity for Selected Disciplines (COMP, ENGI, MEDI and PHYS).

		DEA and NDEA Models			
COMP	P/NA	1.1	1.2	2.1	2.2
P/NA	1.000				
1.1	0.532	1.000			
1.2	0.389	0.635	1.000		
2.1	0.417	0.314	0.262	1.000	
2.2	0.229	0.104	0.416	0.719	1.000
ENGI	P/NA	1.1	1.2	2.1	2.2
P/NA	1.000				
1.1	0.545	1.000			
1.2	0.437	0.646	1.000		
2.1	0.386	0.430	0.337	1.000	
2.2	0.139	0.222	0.555	0.655	1.000
MEDI	P/NA	1.1	1.2	2.1	2.2
P/NA	1.000				
1.1	0.605	1.000			
1.2	0.548	0.805	1.000		
2.1	0.544	0.413	0.418	1.000	
2.2	0.350	0.279	0.502	0.729	1.000
PHYS	P/NA	1.1	1.2	2.1	2.2
P/NA	1.000				
1.1	0.536	1.000			
1.2	0.165	0.371	1.000		
2.1	0.390	0.382	0.235	1.000	
2.2	−0.032	−0.033	0.452	0.619	1.000

**Figure A1:**
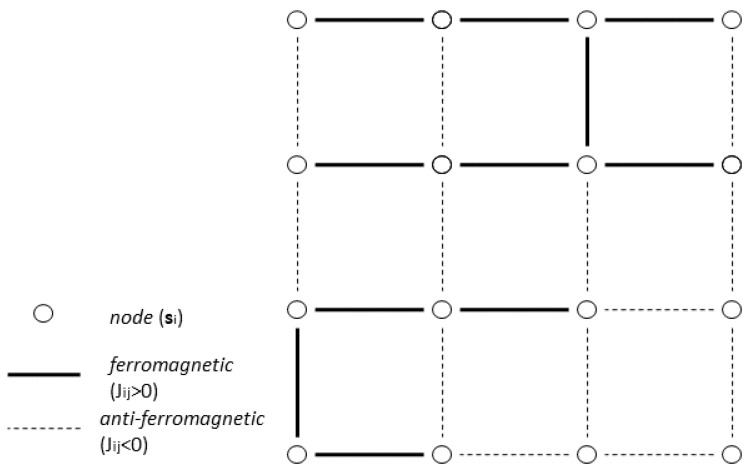
Illustration of an Ising Model. $J_{ij}>0$ correspond to ferromagnetic couplings; $J_{ij}<0$ correspond to anti-ferromagnetic couplings.

## Abbreviations

The following abbreviations are used in this manuscript:

DEA	Data Envelopment Analysis
NDEA	Network Data Envelopment Analysis

## Appendix A. The Ising Spin Glass Model

The Ising spin glass model is made up of a lattice, where each node of the lattice is associated with a vector variable $s_{i}$ at the *i*-th site in the *D*—dimensional space, that represents the spin of a particle. See Figure A1 for an illustration.

Using the relevant Ising and thermodynamic terminology, we can develop a simple spin model to describe the stationary states of our system. For a given couple *i* and *j* the ‘energy’ unit is given by

(A1) $$J_{ij}s_{i}\cdot s_{j},$$

whereas the Hamiltonian is

(A2) $$H=-\frac{1}{2}\beta \sum_{ij=1}^{N}J_{ij}s_{i}\left(t\right)\cdot s_{j}\left(t\right)-\sum_{i=1}^{N}s_{i}\left(t\right)\cdot h_{i}.$$

The parameters of the system are the intensity of the external magnetic field $h_{i}$, the pairwise interactions $J_{ij}$, $\beta ={\left(k_{B}T\right)}^{-1}$. The Hamiltonian in (A2) describes an Ising model, originally introduced to study the behavior of ferromagnetic systems. The Ising model, when used in the study of different systems, e.g., productivity network, can thus also account for a site-independent weight, β, and external biases, $h_{i}$. For the sake of simplicity we fix here β=1 and $h_{i}=0$, ∀i∈(1,N). $J_{ii}=0$, $J_{ij}=J_{ji}$. The total energy of the system is E=<H>. If the system is in equilibrium at a given ‘temperature,’ *T*, then the energy distribution of the units follows the Boltzmann law, given by

(A3) $$F\left(E\right)=\frac{1}{Z}e^{-E/k_{B}T},$$

where *Z* is the partition function introduced in Section 4.2. The component $e^{-E/k_{B}T}$ is known as the Boltzmann factor and $k_{B}$ is Boltzmann’s constant.

The model assumes ergodicity of the system, i.e., the time average of functions on our spin variables equals the average of these same functions over their probability distributions. This hypothesis is commonly assumed for processes that involve human systems, including time series econometrics.

## Appendix B. Pseudo-Likelihood Approach to Inverse Inference Problem

We report in the following specifics of the objective function used in the optimization algorithm to infere the set of parameters {J}, i.e., the Pseudo-Log-Likelihood function, and of its gradient. The fact that the gradient of the Log-Pseudo-Likelihood function can be calculated exactly makes the computational solution of the inference problem faster and more reliable. By relying on Equations (13) and (14), the expression of the Log-Pseudo-Likelihood function is obtained once one has an explicit expression for the local Hamiltonian, $H_{i}\left(s_{i}|\left\{s_{\setminus i}\right\}\right)=-s_{i}\cdot \left[\frac{1}{2}\sum_{i\neq j}^{1,N}J_{ij}s_{j}\right]$ and for the local partition function, $Z_{i}=\sum_{\left\{s_{i}\right\}}e^{-H_{i}\left(s_{i}|\left\{s_{\setminus i}\right\}\right)}$. We define $A_{i}=\frac{1}{2}\sum_{j}^{1,N}J_{ij}s_{j}$, thus

(A4) $$\begin{array}{c} H_{i}\left(s_{i}|\left\{s_{\setminus i}\right\}\right)=-s_{i}\cdot A_{i} \end{array}$$

and

(A5) $$\begin{array}{c} Z_{i}=\sum_{\left\{s_{i}\right\}}e^{s_{i}\cdot A_{i}}\propto \int_{-1}^{1}ds_{i}e^{s_{i}\cdot A_{i}}=\prod_{\gamma =1}^{D}\int_{-1}^{1}ds_{i}^{\left(\gamma \right)}e^{s_{i}^{\left(\gamma \right)}A_{i}^{\left(\gamma \right)}}=\prod_{\gamma =1}^{D}\frac{2\;\;\sinh(A_{i}^{\gamma })}{A_{i}^{\gamma }}. \end{array}$$

The proportionality constant in Equation (A5), equal to the inverse of the total number of all possible $s_{i}$ configurations, does not influence the following derivations and it will be not explicitly considered. The sum $\sum_{\left\{s_{i}\right\}}e^{-s_{i}\cdot A_{i}}$ in Equation (A5) has been calculated by approximating the discrete variables with continuous variables, whose values can continuously vary in the interval [−1,1]. The Pseudo-Log-Likelihood function takes thus the expression

(A6) $$\lambda \left(\left\{J\right\}\right)=\sum_{t=1}^{T}\sum_{i=1}^{N}[s_{i}\left(t\right)\cdot A_{i}\left(t\right)-\sum_{\gamma =1}^{D}\log\left(\frac{2\;\;\sinh(A_{i}^{\left(\gamma \right)}\left(t\right))}{A_{i}^{\left(\gamma \right)}\left(t\right)}\right)]+\mathrm{const}.$$

The gradient of the Log-Pseudo-Likelihood function is given in Equation (15). To calculate the gradient of the Pseudo-Log-Likelihood with respect to the set of parameters $J_{ij}$ we thus need to calculate the quantity $<s_{i}\cdot s_{j}{>}_{i,\{J\}}$. It is

(A7) $$\begin{array}{c} <s_{i}\cdot s_{j}{>}_{i,\{J\}}=\frac{\sum_{\left\{s_{i}\right\}}s_{i}\cdot s_{j}e^{-H_{i}\left(s_{i}|\left\{s_{\setminus i}\right\}\right)}}{\sum_{\left\{s_{i}\right\}}e^{-H_{i}\left(s_{i}|\left\{s_{\setminus i}\right\}\right)}}=\\ \;\;\;\;\;\;\;\;\;\frac{1}{Z_{i}}s_{j}\cdot \int_{-1}^{1}ds_{i}s_{i}e^{s_{i}\cdot A_{i}}=\frac{1}{Z_{i}}\sum_{\gamma =1}^{D}\prod_{\alpha =1}^{D}s_{j}^{\left(\gamma \right)}\int_{-1}^{1}ds_{i}^{\left(\alpha \right)}s_{i}^{\left(\gamma \right)}e^{s_{i}^{\left(\alpha \right)}A_{i}^{\left(\alpha \right)}}=\\ \;\;\;\;\;\;\;\;\;\;\;\;\frac{1}{Z_{i}}\sum_{\gamma =1}^{D}s_{j}^{\left(\gamma \right)}\left[\prod_{\alpha \neq \gamma }^{1,D}\frac{2}{A_{i}^{\left(\alpha \right)}}\sinh A_{i}^{\left(\alpha \right)}\right]\frac{2}{{\left[A_{i}^{\left(\gamma \right)}\right]}^{2}}\left(A_{i}^{\left(\gamma \right)}\cosh A_{i}^{\left(\gamma \right)}-\sinh A_{i}^{\left(\gamma \right)}\right). \end{array}$$

By rephrasing the expression of $Z_{i}$ reported in Equation (A5) we obtain

$Z_{i}\propto \frac{2\;\;\sinh(A_{i}^{\left(\gamma \right)})}{A_{i}^{\left(\gamma \right)}}\prod_{\alpha \neq \gamma }^{1,D}\frac{2\;\;\sinh(A_{i}^{\left(\alpha \right)})}{A_{i}^{\left(\alpha \right)}}$. Inserting this latter expression in Equation (A7), we get

$$\begin{array}{c} <s_{i}\cdot s_{j}{>}_{i,\{J\}}\propto \sum_{\gamma =1}^{D}s_{j}^{\left(\gamma \right)}\frac{2}{{\left(A_{i}^{\left(\gamma \right)}\right)}^{2}}\left(A_{i}^{\left(\gamma \right)}\cosh\left(A_{i}^{\left(\gamma \right)}\right)-\sinh\left(A_{i}^{\left(\gamma \right)}\right)\right)\frac{A_{i}^{\left(\gamma \right)}}{2\;\;\sinh(A_{i}^{\left(\gamma \right)})}=\;\;\;\;\;\;\;\;\;\\ \sum_{\gamma =1}^{D}s_{j}^{\left(\gamma \right)}\left[\frac{1}{\tanh\left(A_{i}^{\left(\gamma \right)}\right)}-\frac{1}{A_{i}^{\left(\gamma \right)}}\right], \end{array}$$

and finally (the proportionality constant for $Z_{i}\left(\left\{J\right\}\right)$ and $<s_{i}\cdot s_{j}{>}_{i,\{J\}}$ is the same)

(A8) $$\begin{array}{c} \frac{\partial }{\partial J_{ij}}\lambda \left(\left\{J\right\}\right)=\frac{1}{2}T\left[Q_{ij}-\frac{1}{T}\sum_{t=1}^{T}\sum_{\gamma =1}^{D}s_{j}^{\left(\gamma \right)}\left(t\right)\left[\frac{1}{\tanh\left(A_{i}^{\left(\gamma \right)}\left(t\right)\right)}-\frac{1}{A_{i}^{\left(\gamma \right)}\left(t\right)}\right]\right]. \end{array}$$

To deal with a lower number of parameters in place of maximizing the Log-Pseudo-Likelihood function, given by the sum of the single-node Log-Pseudo-Likelihood functions (Equation (14)), each single-node Pseudo-Log-Likelihood function is maximized. Since the couplings in the Ising model should be symmetric the final estimate of the $J_{ij}$ parameter is obtained by taking the average $(J_{ij}+J_{ji})/2$.

## Appendix C. Full Scopus 16 Data Summary

**Table A1:** Summary Statistics Performance Variables, by Discipline (ASJC code) (53 Countries, 16 Disciplines, 1996–2012, 901 Observations Each Discipline).

Discipline	Mean	Std. Dev.	Min	Max
AGRI (11)				
Articles	2448.1	4565.2	29	41,261
Highly Cited	328.5	772.9	0	10,068
Number of Authors	4906.4	9635.7	54	97,432
BIOC (13)				
Articles	4357.6	9723.4	44	85,295
Highly Cited	529.1	1572.1	0	15,480
Number of Authors	10,499.4	23,931.7	95	229,139
CENG (15)				
Articles	1211.6	2488.6	11	22,203
Highly Cited	155.0	333.2	0	3557
Number of Authors	2617.4	5788.1	26	65,580
CHEM (16)				
Articles	2686.3	4983.4	31	46,252
Highly Cited	305.0	712.5	0	6460
Number of Authors	5147.5	10,292.8	55	111,789
COMP (17)				
Articles	2727.5	7315.9	7	80,276
Highly Cited	355.6	1013.9	0	10,123
Number of Authors	4161.6	11,548.7	11	128,273
EART (19)				
Articles	1628.3	3360.1	15	24,836
Highly Cited	239.8	582.2	0	4862
Number of Authors	2558.2	5,774.8	20	50,348
ENER (21)				
Articles	635.7	1625.8	3	16,685
Highly Cited	85.0	187.4	0	1974
Number of Authors	1360.0	3771.0	6	47,669
ENGI (22)				
Articles	5433.7	14,078.9	31	156,349
Highly Cited	739.5	1804.5	0	19,830
Number of Authors	8981.8	23,456.8	54	293,605
ENVI (23)				
Articles	1367.7	2827.3	18	22,742
Highly Cited	177.4	400.2	0	3657
Number of Authors	2806.4	6034.3	41	52,584
IMMU (24)				
Articles	1164.6	2406.0	16	18,650
Highly Cited	143.2	395.4	0	3597
Number of Authors	3141.2	6630.4	38	57,748
MATE (25)				
Articles	2751.3	5835.9	22	58,492
Highly Cited	330.5	745.3	0	6939
Number of Authors	4982.2	11,305.5	27	132,822
MATH (26)				
Articles	1804.3	3773.5	7	32,690
Highly Cited	253.9	616.4	0	7565
Number of Authors	2588.5	6174.5	12	63,005
MEDI (27)				
Articles	7672.4	17,175.3	95	165,181
Highly Cited	1,027.9	2929.3	0	28,743
Number of Authors	15,773.2	34,286.3	211	351,702
NEUR (28)				
Articles	968.8	2389.3	2	20,520
Highly Cited	115.8	360.8	0	3340
Number of Authors	2284.5	5636.0	4	53,241
PHAR (30)				
Articles	1103.2	2285.4	12	17,750
Highly Cited	134.6	336.2	0	3385
Number of Authors	2959.7	6299.1	22	53,288
PHYS (31)				
Articles	4396.5	8271.2	19	58,244
Highly Cited	559.9	1208.5	0	10,591
Number of Authors	6946.0	14,391.0	26	127,209
